# Acute pancreatitis induced by combination chemotherapy used for the treatment of acute myeloid leukemia: A case report

**DOI:** 10.1097/MD.0000000000021848

**Published:** 2020-08-28

**Authors:** Qiu-Jin Yang, Jie Zheng, Fu-Tao Dang, Yue-Meng Wan, Jing Yang

**Affiliations:** Department of Gastroenterology, The Second Affiliated Hospital of Kunming Medical University, Kunming, Yunnan Province, China.

**Keywords:** chemotherapeutic agents, diagnosis, drug-induced pancreatitis, pathogenesis, risk factors, treatment principles

## Abstract

**Rationale:**

Drug-induced pancreatitis (DIP) is a kind of acute pancreatitis with a relatively low incidence. There are many cases of acute pancreatitis (AP) caused by chemotherapeutic agents that have been reported. However, few reports focus on the combination of chemotherapeutic agents that induce acute pancreatitis. This article aims to retrospectively analyze a case of DIP and to explore the relationship between chemotherapeutic agents and acute pancreatitis.

**Patient concerns:**

Here, we report a 35-year-old Chinese female patient who was diagnosed as acute myeloid leukemia with BCR/ABL expression. After induction chemotherapy of daunorubicin and cytarabine, bone marrow aspiration showed: Acute myeloid leukemia-not relieved (AML-NR). Then the regimen of homoharringtonine, cytarabine and dasatinib was started. The patient developed abdominal pain on the 14th day of chemotherapy. Laboratory tests showed elevated serum amylase (AMY) and lipase (LIPA). Computed tomography (CT) of the abdomen revealed a swollen pancreas with blurred edges and thickened left prerenal fascia.

**Diagnosis:**

The patient was diagnosed as DIP by the symptoms of upper abdominal pain and the change of CT images. Other common causes of AP were excluded meanwhile.

**Interventions:**

The chemotherapy was stopped immediately. And after fasting, fluid infusion and inhibiting the secretion of the pancreas, the symptoms were relieved.

**Outcomes:**

DIP relapsed when the regimen of aclacinomycin + cytarabine + G-CSF + dasatinib regimen (G-CSF (400ug/day, day 1 to 15), cytarabine (30 mg/day, day 2 to 15), aclacinomycin (20 mg/day, day 2 to 5)and dasatinib (140 mg/day, continuously)) was given, and was recovered after treatment for AP was performed.

**Lessons:**

To choose the best treatment plan for patients, clinicians should raise awareness of DIP, and should know that chemotherapeutic agents can induce pancreatitis and the combination of chemotherapeutic agents may increase the risk of drug-induced pancreatitis.

## 1 Introduction

Acute pancreatitis is a kind of inflammatory injury including pancreatic edema, hemorrhage and necrosis caused by self-digestion of pancreatic tissue. And acute pancreatitis can be caused by various causes. The most common causes include: biliary tract disease, alcohol, pancreatic duct obstruction, disease of the descending duodenum, etc. Drug-induced pancreatitis is relatively rare. At present, there are few studies on drug-induced pancreatitis, mainly based on case reports.^[1]^ However, as more and more drugs are used in clinical practice, the incidence of drug-induced pancreatitis is also relatively increased. Chemotherapeutic agents have been reported repeatedly to cause AP, and the mechanism is still unclear. Combination of chemotherapeutic agents may increase the incidence of DIP. DIP is a diagnosis of exclusion, and it is difficult to determine the causal relationship between the drug and pancreatitis without a rechallenge test.^[2]^ The main point of the treatment for DIP is to stop suspicious drugs, and other therapies are the same as general pancreatitis.^[3]^ Many of the reported cases of pancreatitis are caused by the clear use of a single drug, and few are caused by drug combination or interaction. Therefore, we retrospectively analyzed the course, laboratory examination and imaging changes of a case of acute pancreatitis caused by the combination of chemotherapeutic agents, to deepen the understanding of the risk factors, pathogenesis, diagnosis and treatment principles of DIP via reviewing the literature, and to help clinicians to choose medications. Written informed consent was obtained from the patient for publication of this case report and accompanying images.

## 2 Case presentation

**Figure 1:**
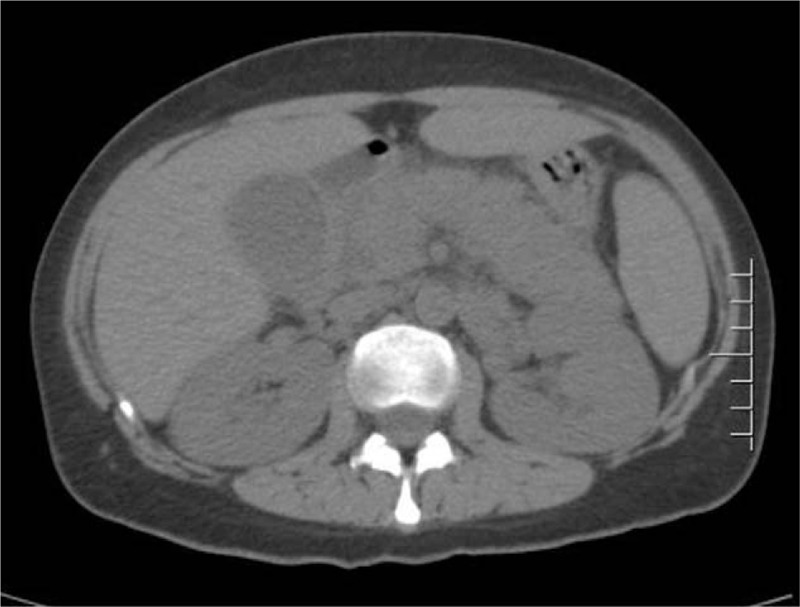
CT of the abdomen revealed a swollen pancreas with blurred edges and thickened left prerenal fascia, which suggested the diagnosis of AP. CT = Computed tomography, AP = Acute pancreatitis.

**Figure 2:**
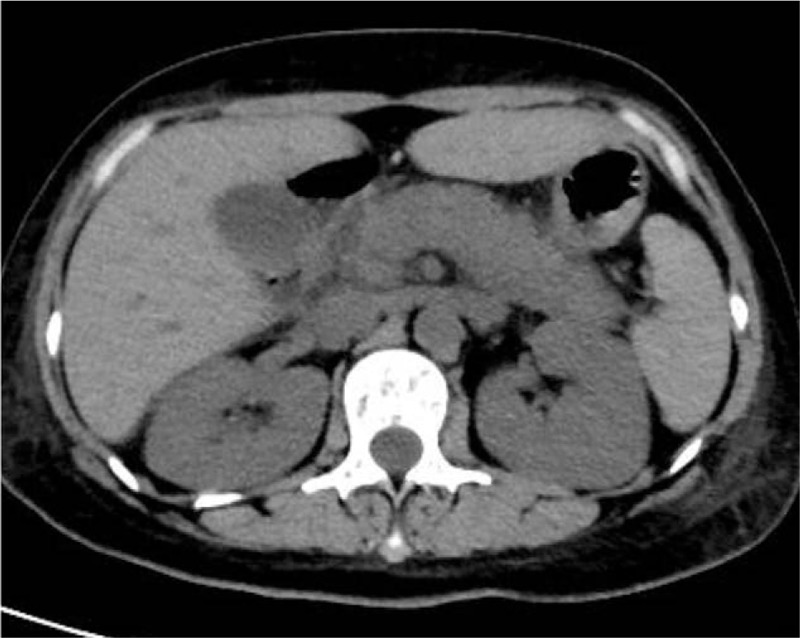
CT of the abdomen showed a swollen pancreas. CT = Computed tomography.

A 35-year-old woman presented with swelling of the right upper gums, without gum pain, bleeding, fever, night sweats, bone pain, weight loss, etc. Blood test showed: white blood cell count (WBC) 13.74 × 10^9^/L, neutrophile cell (NC) 0.82 × 10^9^/L, hemoglobin (Hb) 105 g/L, platelet (PLT) 140 × 10^9^/ L, the primitive cells account for 60%. Then she came to our hospital for further diagnosis and treatment. Bone marrow cytological diagnosis was: acute myeloid leukemia to be classified, with increased immature basophils. Bone marrow biopsy revealed: myeloid hyperplasia is extremely active (>90%), and cells in naive stage increase significantly. Immunohistochemically, BCR-ABL1 p190 was positive. Karyotype analysis of bone marrow showed the presence of 46, XX, t (9; 22) (q34; q11.2). The diagnosis of acute myeloid leukemia with BCR/ABL expression was established. Induced chemotherapy was given, including daunorubicin (90 mg/day, day 5 to 7) and cytarabine (200 mg/day, day 1 to 7) from March 13th to 19th. The patient developed myelosuppression after chemotherapy, including granulocyte deficiency, severe anemia and extremely low platelets, which were alleviated after administration of Granulocyte colony-stimulating factor (G-CSF) and blood transfusion. Bone marrow aspiration on April 4th showed: Acute myeloid leukemia-not relieved (AML-NR). On April 8th, the regimen of homoharringtonine, cytarabine and dasatinib was started: cytarabine (200 mg/day for 7 days), homoharringtonine (3 mg/day, day 1, day 3, day 5 and day 7) and dasatinib (140 mg/day, continuously). The patient developed abdominal pain on the 14th day of chemotherapy. The pancreatic function test was found to be: amylase (AMY) 111U/L (normal range 30–110U/L), lipase (LIPA) 568 U/L (normal range 23–300U/L). Computed tomography (CT) of the abdomen revealed a swollen pancreas with blurred edges and thickened left prerenal fascia (Fig. 1). Diagnosis of acute pancreatitis can be determined by the biochemical test and CT imaging. The use of Dasatinib was stopped immediately. After fasting, fluid infusion and inhibiting the secretion of the pancreas, the symptoms were relieved. The patient returned to our hospital because of fever on May 10th, 2019. After exclusion of the contraindications of chemotherapy, chemotherapeutic regimen of the aclacinomycin, cytarabine, G-CSF and dasatinib was given including G-CSF (400ug/day, day 1 to 15), cytarabine (30 mg/day, day 2 to 15), aclacinomycin (20 mg/day, day 2 to 5) and dasatinib (140 mg/day, continuously). The patient developed abdominal pain and fever on May 29th, 2019. Emergency pancreatic function test showed increased trypsin: AMY 134U/L (normal range 30-110U/L), LIPA 520U/L (normal range 23–300U/L). CT of the abdomen showed a swollen pancreas (Fig. 2). We considered it a relapse of pancreatitis. The use of dasatinib was stopped again and treatment for pancreatitis was given until the symptoms were relieved and pancreatic function returned to normal.

## 3 Discuss

DIP is a kind of acute pancreatitis with a relatively low incidence. At present, the incidence of DIP is mostly from random case reports, but not all case reports clearly describe the dose, frequency, and time course from drug delivery to onset, and some cases have not even strictly ruled out other causes of acute pancreatitis. Due to ethical issues, few cases can complete the rechallenge test. Nitsche et al^[1]^ believe that the overall incidence of drug-induced pancreatitis may be between 0.1% and 2%. The incidence of drug-induced pancreatitis in the study of Vinklerová et al^[2]^ is as high as 5.3% (9/170) however, which may be attributed to the small number of cases, or because most cases cannot be diagnosed by the rechallenge test and lead to over-diagnosis.

There are no unified diagnostic criteria for drug-induced pancreatitis at home and abroad so far. Jiaming Qian and Shujun Wang^[3]^ proposed that the diagnostic criteria for DIP are:

(1) meeting the diagnostic criteria for acute pancreatitis;
(2) excluding other common causes of acute pancreatitis;
(3) history of medication;
(4) the time from drug delivery to onset is consistent with the latency reported in most literatures;
(5) the improvement of pancreatitis symptoms and the decrease of pancreatic enzyme after withdrawal;
(6) after re-medication clinical manifestations reappeared and pancreatic enzymes increased (rechallenge test positive).

It is worth noting that in the diagnosis of DIP, it is necessary to carefully ask and find out the history, to rule out common causes of acute pancreatitis such as cholelithiasis, heavy drinking, hyperlipidemia, autoimmune pancreatitis, pancreatic cancer, abdominal injury and the history of surgery, etc. If the condition allows and the patient's consent is obtained, the same drug can be used again if necessary. If clinical symptoms, pancreatic enzyme elevation increase and/or imaging changes occur again, suggesting that the rechallenge test is positive, which indicates the diagnosis of DIP.

The WHO database lists a total of 525 drugs that may cause acute pancreatitis. But there are currently only about 30 drugs that cause acute pancreatitis for certain. Drugs that are now clearly associated with pancreatitis are listed in Table 1.^[4]^

**Table 1:**
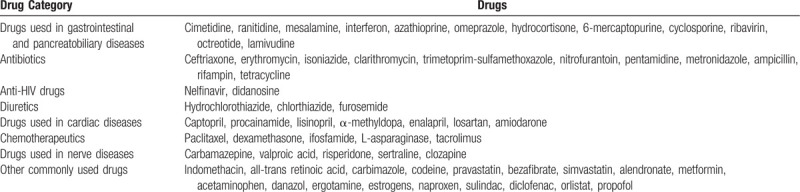
Drugs definitely associated with acute pancreatitis according to Ksiadzyna^[4]^.

The pathogenesis of drug-induced pancreatitis is still unclear. Yanar et al^[5]^ pointed out that the hypothesis of mechanism include immune-mediated inflammatory response, direct cytotoxicity, pancreatic duct stenosis, small arterial thrombosis and metabolic effects, etc. Studies by Badalov et al^[6]^ have shown that drugs such as acetaminophen, erythromycin, and carbamazepine induce DIP through direct toxic damage to the pancreas by active metabolites, and this response is associated with overdose. Pancreatitis caused by most drugs is not related to the dose of the drug, so most drug-induced pancreatitis is caused by an idiosyncratic reaction instead, that is, an unpredictable reaction that occurs within the normal dose of the drug.^[3]^ Some side effects of drugs can lead to hypercalcemia, hyperlipidemia or increased pancreatic juice viscosity, which is exactly the pathogenesis of acute pancreatitis.^[3,7]^

Cytarabine mainly acts on pyrimidine antimetabolites in the S proliferative phase of cells. It inhibits DNA synthesis and interferes with cell proliferation. It widely uses in leukemia, especially for acute myeloid leukemia. There have been reports of cases of cytarabine-associated pancreatitis, but few of them have accomplished the rechallenge test, and the pathogenesis is still not very clear. Dasatinib is a polytyrosine kinase inhibitor, mainly used in the treatment of adult patients with all stages of chronic myelogenous leukemia who is imatinib-resistant or imatinib-intolerable and the treatment of adult patients resistant or intolerant to other therapies with Philadelphia chromosome-positive acute lymphoblastic leukemia. Serpa et al^[8]^ reported a case report of acute pancreatitis after the use of dasatinib, the mechanism of TKIs(tyrosine kinase inhibitors)-induced pancreatitis is still speculation, but the reason proposed by Plandari et al^[9]^ may be the inhibition of c-ABL. This inhibition may interfere with the molecular mechanism that regulates apoptosis and leads to pancreatic damage. Or may be due to the indirect effects of drugs on the release of calcium ions from the intracellular acinar pool, thereby regulating pancreatic exocrine function and possibly increasing the deposition of intracellular fatty acids in pancreatic acinar cells, which interfere with the exocytosis process. However, reports of pancreatitis caused by TKIs are more common in imatinib and nilotinib, and dasatinib is relatively rare. Case reports of acute pancreatitis induced by homoharringtonine^[10]^ and aclacinomycin^[11]^ have also been published, but lack of relevant mechanisms.

In this case, the patient didn’t develop any sign of acute pancreatitis when she underwent the regimen of daunorubicin and cytarabine at the stage of induced chemotherapy (daunorubicin(90 mg/day, day 5 to 7) and cytarabine (200 mg/day, day 1 to day 7)). However, she suffered from acute pancreatitis on the 14th day when she underwent the chemotherapy regimen of homoharringtonine, cytarabine and dasatinib (cytarabine(200 mg/day for 7 days), homoharringtonine (3 mg/day, day 1, day 3, day 5 and day 7) and dasatinib(140 mg/day, continuously)). After her acute pancreatitis was cured, she developed acute pancreatitis again on the 15th day when she underwent the chemotherapy regimen of aclacinomycin, cytarabine, G-CSF and dasatinib (G-CSF (400ug/day, day 1 to 15), cytarabine (30 mg/day, day 2 to 15), aclacinomycin (20 mg/day, day 2 to 5) and dasatinib(140 mg/day, continuously)). Other common causes of acute pancreatitis such as cholelithiasis, alcohol, and hyperlipidemia have been ruled out by making a detailed inquiry of her medical history and relevant auxiliary examinations. Therefore, the diagnosis of DIP was established. And both episodes of pancreatitis were cured by symptomatic and supportive treatment for acute pancreatitis such as fasting, anti-infection, inhibition of gastric acid and inhibition of pancreatic enzyme secretion. Since all the four chemotherapeutic agents used in this patient have been reported to be with side effects causing acute pancreatitis, in consideration of the three chemotherapeutic regimens of the patient and the incubation period before acute pancreatitis, cytarabine is most likely the chief culprit in inducing her acute pancreatitis in this case. Cytarabine has been reported to cause acute pancreatitis for many time, in the Badalov 2007 classification criteria,^[5]^ it belongs to class Ia (at least one case reported a positive rechallenge test, and ruled out other causes). McBride et al^[12]^ have reported that cytarabine may cause acute pancreatitis for two reasons:

(1) Time-dependent toxicity;
(2) Combined with other chemotherapeutic agent.

McGrail^[13]^ and others believe that acute pancreatitis induced by cytarabine has nothing to do with the time and dose. In this case, there was also no clear dose-dependent and time-dependent relationship between cytarabine and acute pancreatitis, and it is more likely to be attributed to the combination with other drugs.

There are relatively few reports of acute pancreatitis caused by dasatinib, homoharringtonine, and aclacinomycin. However, the possibility of pancreatitis caused by any other single drugs or the combination of them in this case cannot be completely ruled out. We figured that there was no aclacinomycin in the chemotherapeutic regimen of homoharringtonine, cytarabine and dasatinib at the first time when the patient developed acute pancreatitis, and there was no homoharringtonine in the regimen of the aclacinomycin, cytarabine, G-CSF and dasatinib at the second time, considering that the possibility of the acute pancreatitis induced by these two drugs was relatively low. There was no acute pancreatitis when the patient underwent high-dose cytarabine therapy at the stage of inducing chemotherapy, and since dasatinib was continuously used through the other two stages, it may be another potential cause of DIP. Therefore, it is difficult to clearly prove the causal relationship between any single drug and acute pancreatitis in this case.

We assume that cytarabine combined with some certain drugs may increase the risk of inducing acute pancreatitis, or several combinations of drugs that may cause pancreatitis will increase the incidence of drug-induced pancreatitis. The pathogenesis needs more further research. When selecting the appropriate chemotherapeutic regimen for a patient, the clinician should carefully consider the superposition of the same adverse reactions of the drugs and the interaction between the drugs to avoid using a combination of drugs that may increase the incidence of adverse reactions. At the same time, when clarifying the relationship between a single drug and acute pancreatitis, it is also necessary to consider the additional effects of multiple drugs in order to help clinicians to develop better treatment options.

Due to the complexity of the diagnosis of drug-induced pancreatitis, it often leads to misdiagnosis or missed diagnosis. Tenner^[14]^ pointed out that clinicians were overly dependent on case reports and ignored randomized clinical trials and large pharmacology-epidemiology investigations, leading to confusion about drug-induced acute pancreatitis and idiopathic pancreatitis. He believes that drug-induced pancreatitis may account for less than 1% of cases of pancreatitis, and may be extremely rare in patients who do not take significant medications. Nearly one-third of pancreatitis are described as idiopathic pancreatitis because of its unknown etiology. Therefore, it is a more appropriate choice to diagnose it as idiopathic pancreatitis when the diagnosis of drug-induced pancreatitis is not clear.^[14]^

In view of the idiopathic and susceptibility characteristics of drug-induced pancreatitis, we can prevent it by the following ways. First of all, we should fully understand the drugs that may cause pancreatitis. Secondly, we should recognize the susceptible population of drug-induced pancreatitis. Balani et al^[15]^ pointed out that the risk factors of drug-induced pancreatitis include:

(1) children
(2) women
(3) elderly patients taking multiple drugs
(4) CD4^+^T-cells count for <200 cells/mm3 of advanced AIDS patients
(5) inflammatory bowel disease (especially Crohn's disease)
(6) patients who use tumor chemotherapeutic drugs.

Particular attention should be paid to the possibility of developing drug-induced pancreatitis when using related drugs in these groups. For the treatment of drug-induced pancreatitis, the most important thing is to stop using suspicious drugs, and other therapies are the same as general pancreatitis.^[3,7]^ To avoid re-use of the same drug as much as possible. If considering that the benefits for the patient are greater than the side effects (pancreatitis) when it is necessary to use a drug, we can use a possible alternative or change the dose and method to minimize the incidence of side effects.

## 4 Summary

With the continuous development of new drugs, more and more drugs have been used in clinical practice, and drugs reported causing acute pancreatitis are also increasing. Among them, chemotherapeutic drugs can induce pancreatitis and the combination of chemotherapeutic drugs may increase the risk of drug-induced pancreatitis, which should cause enough attention of clinicians. The specific incidence of pancreatitis caused by chemotherapeutic drugs and the combination of chemotherapeutic agents that cause DIP needed further study at present.

## Acknowledgments

The authors thank to the contribution of their colleagues and institution.

## Author contributions

**Conceptualization:** Qiujin Yang, Jie Zheng.

**Data curation:** Qiujin Yang, Jie Zheng.

**Investigation:** Qiujin Yang, Jie Zheng, Fu-Tao Dang.

**Methodology:** Qiujin Yang, Jie Zheng.

**Project administration:** Jing Yang.

**Resources:** Fu-Tao Dang.

**Supervision:** Jing Yang.

**Validation:** Yue-Meng Wan, Jing Yang.

**Visualization:** Yue-Meng Wan, Jing Yang.

**Writing – original draft:** Qiujin Yang, Jie Zheng.

**Writing – review & editing:** Qiujin Yang, Jie Zheng, Jing Yang.

## References

[1] Nitsche CJ Jamieson N Lerch MM Drug induced pancreatitis. Best Pract Res Clin Gastroenterol 2010;24:143 55. 20227028 10.1016/j.bpg.2010.02.002

[2] Vinklerová I Procházka M Procházka V Incidence, severity, and etiology of drug-induced acute pancreatitis. Dig Dis Sci 2010;55:2977 81. 20499176 10.1007/s10620-010-1277-3

[3] Jiaming Qian Shujun Wang Diagnosis and treatment of drug-induced pancreatitis. Lin Chuang Gan Dan Bing Za Zhi 2014;30:722 5.

[4] Ksiadzyna D Drug-induced acute pancreatitis related to medications commonly used in gastroenterology. Eur J Intern Med 2011;22:20 5. 21238888 10.1016/j.ejim.2010.09.004

[5] Yanar F Agcaoglu O Sarici IS Clinical challenges in drug induced pancreatitis: Presentation of two cases and review of the literature. Int J Surg Case Rep 2013;4:708 10. 23810919 10.1016/j.ijscr.2013.02.025 PMC3710901

[6] Badalov N Baradarian R Iswara K Drug-induced acute pancreatitis: an evidence-based review. Clin Gastroenterol Hepatol 2007;5:648 61. quiz 644. 17395548 10.1016/j.cgh.2006.11.023

[7] Chengliang Cao Bei Sun Gang Wang Research progress in drug-induced pancreatitis. Zhongguo Shi Yong Wai Ke Za Zhi 2016;36:1345 7.

[8] Serpa M Sanabani SS Bendit I Efficacy and tolerability after unusually low doses of dasatinib in chronic myeloid leukemia patients intolerant to standard-dose dasatinib therapy. Clin Med Insights Oncol 2010;4:155 62. 21234296 10.4137/CMO.S6413 PMC3018898

[9] Palandri F Castagnetti F Soverini S Pancreatic enzyme elevation in chronic myeloid leukemia patients treated with nilotinib after imatinib failure. Haematologica 2009;94:1758 61. 19608673 10.3324/haematol.2009.010496 PMC2791949

[10] Tang J Liu Y Chen J Homoharringtonine as a backbone drug for the treatment of newly diagnosed pediatric acute myeloid leukemia: a report from a single institution in China. Int J Hematol 2011;93:610 7. 21509439 10.1007/s12185-011-0837-4

[11] Adachi S Akiyama Y Takimoto T Aclarubicin-related pancreatitis in a child with AML. Rinsho Ketsueki 1988;29:385 8. 3165144

[12] McBride CE Yavorski RT Moses FM Acute pancreatitis associated with continuous infusion cytarabine therapy: a case report. Cancer 1996;77:2588 91. 8640710 10.1002/(SICI)1097-0142(19960615)77:12<2588::AID-CNCR24>3.0.CO;2-N

[13] McGrail LH Sehn LH Weiss RB Pancreatitis during therapy of acute myeloid leukemia: cytarabine related? Ann Oncol 1999;10:1373 6. 10631468 10.1023/a:1008342320532

[14] Tenner S Drug induced acute pancreatitis: does it exist? World J Gastroenterol 2014;20:16529 34. 25469020 10.3748/wjg.v20.i44.16529 PMC4248195

[15] Balani AR Grendell JH Drug-induced pancreatitis: incidence, management and prevention. Drug Saf 2008;31:823 37. 18759507 10.2165/00002018-200831100-00002

